# Confocal soft X-ray scanning transmission microscopy: setup, alignment procedure and limitations

**DOI:** 10.1107/S1600577514022322

**Published:** 2015-01-01

**Authors:** Andreas Späth, Jörg Raabe, Rainer H. Fink

**Affiliations:** a Physikalische Chemie II and ICMM, Friedrich-Alexander Universität Erlangen-Nürnberg (FAU), Egerlandstraße 3, 91058 Erlangen, Germany; b Swiss Light Source, Paul Scherrer Institut, 5232 Villigen, Switzerland; c CENEM, Friedrich-Alexander Universität Erlangen-Nürnberg (FAU), Egerlandstraße 3, 91058 Erlangen, Germany

**Keywords:** STXM, soft X-ray microscopy, confocal microscopy, zone plate

## Abstract

Zone-plate-based scanning transmission soft X-ray microspectroscopy (STXM) is a well established technique for high-contrast imaging of sufficiently transparent specimens (*e.g.* ultrathin biological tissues, polymer materials, archaeometric specimens or magnetic thin films) with spatial resolutions in the regime of 20 nm and high spectroscopic or chemical sensitivity. However, due to the relatively large depth of focus of zone plates, the resolution of STXM along the optical axis so far stays unambiguously behind for thicker X-ray transparent specimens. This challenge can be addressed by the implementation of a second zone plate in the detection pathway of the beam, resulting in a confocal arrangement. Within this paper a first proof-of-principle study for a confocal STXM (cSTXM) and an elaborate alignment procedure in transmission and fluorescence geometry are presented. Based on first confocal soft X-ray micrographs of well known specimens, the advantage and limitation of cSTXM as well as further development potentials for future applications are discussed.

## Abstract 2

A conventional STXM setup has been upgraded with a second micro zone plate and aligned to confocal geometry. Two confocal geometries (in-line and off-axis) have been evaluated and a discussion on prospects and limitations is presented.

## 1. Introduction   

The most urgent issue in modern microscopy development is the combination of ultra-high resolution with the possibility of imaging in all three dimensions, since the three-dimensional (3D) nanostructure of an investigated specimen has basic influence on its biological, chemical and physical properties. During the last years outstanding progress in visible-light (Hell & Wichmann, 1994 ▸; Dyba & Hell, 2002 ▸; Rittweger *et al.*, 2009 ▸) and X-ray microscopy (Dierolf *et al.*, 2010 ▸; Chao *et al.*, 2012 ▸; Schneider *et al.*, 2012 ▸; Schropp *et al.*, 2012 ▸; Holler *et al.*, 2014 ▸) have pushed the spatial resolution to the regime of 10 nm. So far X-ray microscopy has not yet reached the diffraction limit due to limitations of the available optics. In particular, soft X-ray microscopy offers the possibility of resonant imaging within the water window, thus providing superior chemically selective contrast for various soft-matter specimens without staining at moderate radiation dose (Ade & Hitchcock, 2008 ▸).

Soft X-ray tomography reached a full 3D resolution of 36 nm (Schneider *et al.*, 2010 ▸), and from several coherent techniques resonant Fourier transform holography (FTH) stands out with a lateral resolution of 16 nm reported using synchrotron radiation (Zhu *et al.*, 2010 ▸). Recent holographic studies with free-electron laser illumination point already towards a new benchmark (Wang *et al.*, 2012 ▸). On the other hand these techniques suffer from fundamental drawbacks. The necessity of sample rotation in tomography typically leads to more complicated setups as well as relatively high sampling times (Haddad *et al.*, 1994 ▸; Schroer *et al.*, 2010 ▸; Schneider *et al.*, 2012 ▸) and therefore increased danger of specimen degradation due to radiation damage. Coherent techniques are especially prone to illumination and setup instabilities (Schropp *et al.*, 2012 ▸) as well as optics aberrations (Chabior *et al.*, 2012 ▸).

Very recently it was shown that a standard scanning transmission soft X-ray microspectroscope (STXM) equipped with high-resolution Fresnel zone plates can provide focal stacks of semi-transparent specimens with sub-20 nm resolution laterally and sub-micrometre resolution along the optical axis (Späth *et al.*, 2014*a* ▸). 3D reconstructions are easily generated from those stacks at moderate computation times by a focus measure based algorithm. The key components of this technique are modern zone plates with ultra-small outermost zone widths providing a lateral resolution of about 10 nm and a depth of focus (DOF) of about 500 nm in the soft X-ray regime (Jefimovs *et al.*, 2007 ▸; Vila-Comamala *et al.*, 2009 ▸). This approach intuitively points to the development of a confocal STXM (cSTXM) using a second high-resolution zone plate in the detection pathway and a focus filtering pinhole in its back focal plane. True confocal setups can significantly improve the 3D resolution far beyond the limits of the used optics and increase the imaging quality by background suppression (Sheppard & Choudhury, 1977 ▸; Brakenhoff *et al.*, 1979 ▸). Present confocal X-ray microscopes usually combine focused hard X-ray illumination with fluorescence detection in a 90° geometry by the use of capillary optics (Janssens *et al.*, 1996 ▸; Kanngiesser *et al.*, 2003 ▸). These confocal micro X-ray fluorescence setups have been successfully employed for material science and environmental studies, but the provided 3D resolution is so far limited to several micrometres (Nakano *et al.*, 2011 ▸; Fittschen & Falkenberg, 2011 ▸; Kanngiesser *et al.*, 2012 ▸). Results from confocal full-field TXM in the hard X-ray regime showed a resolution in the micrometre regime, but are so far limited by very large illumination times and detection limits (Takeuchi *et al.*, 2009 ▸, 2010 ▸). cSTXM, however, provides a lateral resolution down to 10 nm according to the present limits in zone-plate technology (Jefimovs *et al.*, 2007 ▸; Vila-Comamala *et al.*, 2009 ▸). In analogy with correspondent confocal laser scanning microscopes (CLSM) (Dixon *et al.*, 1991 ▸) the axial resolution is expected to be worse by a factor of roughly two due to the blurring of the point-spread function (Nasse & Woehl, 2010 ▸). Future resolution limits will depend on the progress in zone plate development and may reach several nanometres during the next few years.

Within this paper we will present a proof-of-concept study based on two possible setups and an elaborate alignment procedure for cSTXM that were developed by implementation of a detection zone plate and focus filtering pinhole to the standard PolLux-STXM (Raabe *et al.*, 2008 ▸) at the Swiss Light Source. In accordance with existing confocal laser microscopy setups (Pawley, 2006 ▸) we investigated an in-line transmission and an off-axis fluorescence detection installation. We will for the first time show experimental results from cSTXM operating in the soft X-ray regime and discuss the limitations of the setups concerning photon flux and detection efficiency. From our results we will conclude strategies for further development of this promising technique.

## 2. Setup and alignment procedure   

The standard STXM setup uses high-brilliance synchrotron radiation light that is focused on the sample by a Fresnel zone plate and allows for high-resolution spectroscopic contrast due to tunable photon energy (Kirz & Rarback, 1985 ▸; Ade *et al.*, 1992 ▸). The sample is raster-scanned with interferometric control (position stability: <3 nm) through the focal spot with degrees of freedom in all three dimensions, while the transmitted photon intensity is recorded using a phosphor screen attached to a photomultiplier tube (PMT) or a photodiode (Kilcoyne *et al.*, 2003 ▸; Raabe *et al.*, 2008 ▸). Two-dimensional images can be recorded with a fast-read-out CCD camera (Raabe *et al.*, 2009 ▸). Since the zone plate generates various diffraction orders, an order-sorting aperture (OSA) close to the sample is used to filter the requested first-order illumination cone. For our first experiments we used zone plates with an outermost zone width *r*_N_ of 25 nm that provide a first-order DOF of approximately 1400–1500 nm in the investigated photon energy regime (applied as wavelength λ in nanometres) according to (Attwood, 1999 ▸)
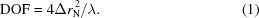
These zone plates do not yet give the best achievable axial resolution, but higher photon rates than 15 or 12.5 nm zone plates, and were therefore better suited for basic tests of the experimental setup.

**Figure 1:**
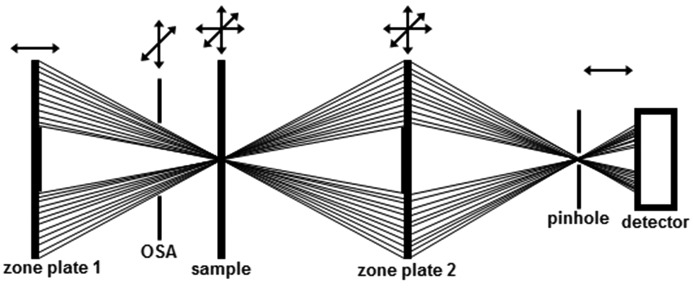
Scheme of cSTXM in transmission geometry. Arrows at the top show the degrees of freedom of the respective scanner stages (pinhole and detector are connected). The distances of the optical elements are not true to scale.

The concept of cSTXM is adopted from conventional CLSM (Dixon *et al.*, 1991 ▸). However, due to special limitations reflection geometries (180°) cannot be realised and thus a second optical element [in our case a Fresnel zone plate (FZP)] is required. We started our investigations with an in-line cSTXM arrangement that is schematically depicted in Fig. 1 ▸. Therefore we had to extend the standard STXM setup with a second zone plate (ZP2) and a pinhole between sample and detector and perfectly centre those on the optical axis. ZP2 is mounted on a three-axis SmarACT stage ensuring high-precision positioning with accuracies better than 20 nm. The *z*-resolution of a confocal setup is strongly influenced by the size of the detection pinhole. Employing the thin-lens equation a sample-to-second-zone-plate distance of some centimetres prevents the need for pinholes smaller than 100 nm (Sheppard & Choudhury, 1977 ▸; Wilson & Carlini, 1987 ▸). Due to the pinhole there is no need for an OSA in the detection path. During the alignment procedure the signal was recorded with a CCD camera (Andor DV860). The pinhole (5 µm diameter for the test setup) was directly glued in front of the scintillator of the camera to achieve very high position stability. The detection area was large enough to move the detector to a position where the pinhole mount was no longer shadowing the photon beam.

**Figure 2:**
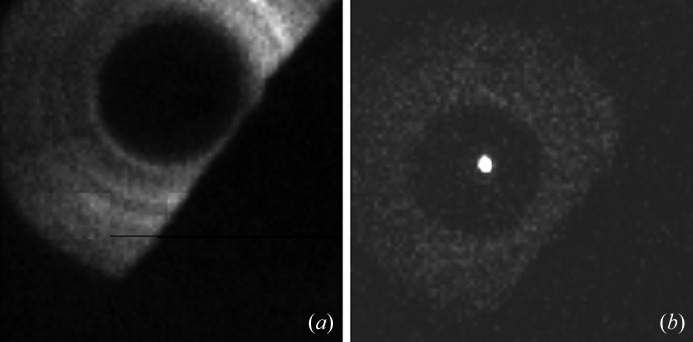
CCD camera records during alignment procedure. (*a*) Standard STXM setup without second zone plate. The first zone plate generates an illumination cone that is divergent behind the sample and hollow due to the central stop. The shadow in the lower right corner is caused by the pinhole mount that was fixed in front of the CCD camera. (*b*) cSTXM with second zone plate in focus. The illumination cone is focused in the optical axis. The bright ring is residual zero light from the second zone plate (no OSA in detection path).

Fig. 2(*a*) ▸ shows a CCD image of this situation without the second zone plate. The detected signal is a projection of the illumination zone plate resulting in a bright ring around a dark disk that represents the central stop. The alignment of the zone plates was performed without sample in the beam path. In the lower right part of the image the pinhole mount is visible as a dark shadow. The correct position of the second zone plate should be roughly estimated by eye when it is installed. After moving it to the estimated position we used the *x*- and *y*-scanners of this zone plate to align it according to the illumination optics. The alignment is perfect when the bright ring is fully visible and homogeneous, while a bright spot is visible in the centre of the dark disk. Note that the bright ring is now much weaker and represents the residual zero-order light from the second zone plate, *i.e.* the not-focused portion. The first-order diffracted light is focused in the bright spot that is usually more of a small ring at the beginning. By scanning the *z*-position of the second zone plate we optimize the focus and try to make the spot as small and bright as possible. The final situation of this step is shown in Fig. 2(*b*) ▸.

The pinhole position was also roughly positioned during installation. It is aligned by scanning the detector *x*- and *y*-stages. At the end of the alignment procedure the focus of the second zone plate has to be optimized. Therefore we scanned its *x*- and *y*-stages consecutively against the *z*-stage and chose the positions at which we obtained the brightest signal. To find and focus a requested sample position the second zone plate should be moved out of the direct beam. The interferometric control ensures that the alignment is maintained after moving it in again. It is important to focus the sample by adjustment of its *z*-position without moving the first zone plate. Otherwise the confocal alignment is lost. We also replaced the CCD camera by the PMT to record the signal in counting mode during scanning. Both detectors can be installed simultaneously, and switching between the detectors can be done by moving the stages to the respective position.

**Figure 3:**
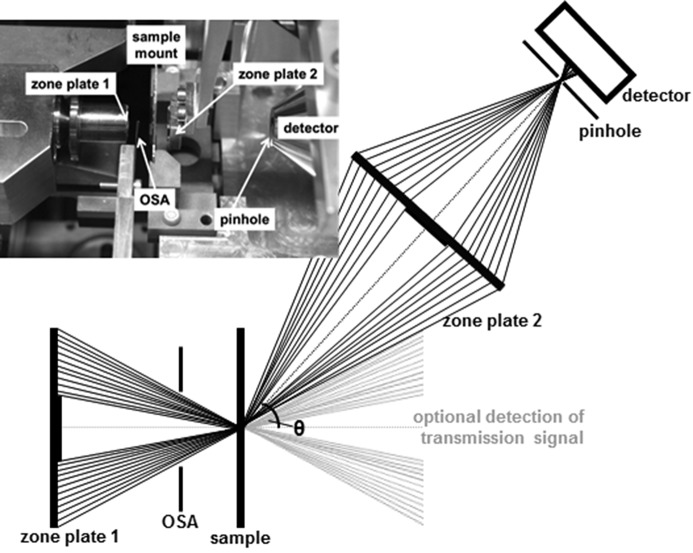
Scheme of cSTXM in fluorescence geometry including a photograph of the setup inside the PolLux chamber (sample far out of focus for better overview). The offset angle of the detection path can be chosen according to setup requirements. In the present study the detection equipment was just slightly moved upwards to miss the direct beam.

For further investigations we modified the setup to an off-axis geometry detecting fluorescence (*cf*. Fig. 3 ▸). Since fluorescence is not directed, the detection angle can be chosen in accordance with setup requirements (5° ≤ θ ≤ 170°), *i.e.* reflectance geometries are also possible for non-transparent samples. In the present study we moved ZP2 and the detector just slightly upwards (θ ≃ 6°) to miss the direct transmitted photon beam. Ideally the detection devices should also be rotated as depicted in Fig. 3 ▸. However, for very small angles the resulting deviation can be neglected as long as we do not explore the ultimate resolution. The first test was performed without pinhole to check for basic fluorescence imaging before pushing the resolution limits. The signal was recorded with an avalanche photodiode, since these detectors provide single-photon sensitivity (Dautet *et al.*, 1993 ▸). One could also consider detecting the transmitted signal by the parallel use of several detectors in future studies. The alignment procedure at the beginning was the same as for the in-line setup. Then ZP2 was moved upwards by 260 µm. The position of the detector was corrected applying Pythagoras’ theorem and has to be adjusted in accordance with the detected count rate. The setup is also shown in a small photograph inset in Fig. 3 ▸. The distances of the optical components as they appear in this image are not suitable for confocal imaging, but adjusted for a better overview.

## 3. Results and discussion   

**Figure 4:**
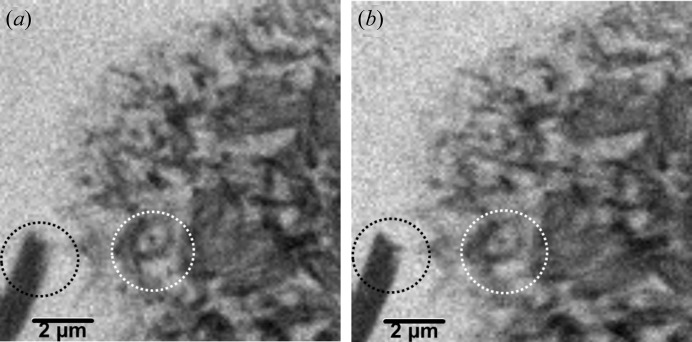
cSTXM micrographs of AgTCNQ crystals from transmission geometry (710 eV, 120 × 120 pixel, 30 ms dwell time). (*a*) Small AgTCNQ crystals lying on a rhombic TCNQ crystal (white circle) are in good focus, while the large AgTCNQ crystal in the lower left corner (black circle) is slightly defocused. (*b*) Sample shift of 4 µm along the optical axis moves the large crystal into proper focus, while the small crystals are slightly defocused.

The first reported micrographs from a cSTXM setup in in-line geometry are shown in Fig. 4 ▸. The investigated samples were AgTCNQ nanocrystals that had been grown from acetonitrile solution in a wet-chemical process on a standard 100 nm-thick Si_3_N_4_ membrane (Silson Ltd, UK) (Rösner *et al.*, 2013 ▸). The micrographs show an array of small crystals that are lying on a large rhombic crystal from residual TCNQ. The TCNQ crystal is hardly visible at the applied illumination energy of 710 eV, since it contains just low-*z* elements and appears therefore almost transparent. On the other hand lower energies in the C *K*-edge regime were not possible due to low focal length and resulting geometrical conflicts within the current setup. In the lower left corner of the micrographs a larger AgTCNQ is found that is lying directly on the membrane. Due to this beneficial condition the two crystal types are not in the same plane perpendicular to the optical axis and should show a deviation in focus. This expectation is confirmed in the cSTXM micrographs. In Fig. 4(*a*) ▸ the small crystals are in proper focus while the larger one is slightly defocused. In Fig. 4(*b*) ▸, which was recorded with a shift of the sample along the optical axis by 4 µm, the situation is reversed. The defocused parts on the micrographs are not filtered so far, since the pinholes used during these basic investigations were still too large to provide a focus filtering effect. Nevertheless the con­focal setup is working in principle.

The first micrographs already reveal an important issue for future cSTXM studies. Also the dwell time was 10–15 times longer than for reasonable STXM images of this specimen, the count rate was low and therefore also the signal-to-noise ratio (SNR) within the micrographs in Figs. 4(*a*) and 4(*b*) ▸ is relatively poor. This finding is not surprising, since zone plates have a first-order diffraction efficiency of about 10% (Attwood, 1999 ▸). Therefore, every additional zone plate reduces the count rate of the setup at least by the factor of ten. The application of a smaller pinhole, however, will strongly increase the imaging quality due to better background suppression. Very long irradiation times, however, increase the risk of beam-induced damage. Radiation chemistry in soft X-ray microscopy has been intensively studied and can be reduced by cryo-techniques (Schneider, 1998 ▸; Beetz & Jacobsen, 2003 ▸; Meents *et al.*, 2010 ▸; Späth *et al.*, 2014*b* ▸).

In the case of fluorescence detection with the off-axis setup the issue of low count rates was more prominent. We were not able to achieve reasonable imaging of various samples at their respective resonant energies, including magnetite-functionalized PVA-based microballoons (Brismar *et al.*, 2012 ▸) (710 eV, Fe *L*α-edge) and Cu deposits (935 eV, Cu *L*α-edge) on Si_3_N_4_ membranes even for very long illumination times. We performed a two-dimensional scan of the detector according to the second zone plate with a pixel dwell time of 1 s. The illuminated sample was a very dense magnetite accumulation on the microballoon sample (Späth *et al.*, 2014*a* ▸). The resulting scan (see Fig. S1 in the supporting information^1^) proves the presence of a fluorescence signal behind the detection zone plate. On the other hand this signal is extremely weak and hardly exceeds the background noise level.

A quantitative estimation of the photon efficiency of our off-axis cSTXM setup is summarized in Table 1 ▸. It shows that the main contribution to the overall efficiency is not derived from the fluorescence yield that is about 10^−2^ for most common metal *L*-edges but from the extremely small detection area (solid angle) covered by the comparably small detection zone plate. Common X-ray fluorescence microscopes use several large-area detectors to cover a larger solid angle and thus detect as many photons as possible (Gianoncelli *et al.*, 2013 ▸). Considering a zone-plate diameter of 240 µm and a distance from the sample of about 25 mm, the zone plate covers roughly a portion of 6 × 10^−6^ of the potentially available sphere around the sample. Thus, the resulting overall photon efficiency of the setup is in the regime of 10^−11^ according to the detector type. This estimation does not take into account self-absorption at the sample that can have further impact on the detected signal. Considering imaging at reasonable recording times in the microsecond regime, even high-flux undulator beamlines may not provide enough photons in the soft X-ray regime [∼10^13^ s^−1^ (Strocov *et al.*, 2010 ▸; Yamamoto *et al.*, 2014 ▸)] to achieve fluorescence images of sufficient quality and SNR.

## 4. Conclusions and outlook   

The concept of scanning confocal microscopy has been employed to allow for confocal imaging in the soft X-ray regime by extension of a conventional STXM with a second FZP in the detection pathway. Thus, limitations from visible-light microscopy in terms of lateral resolution can be overcome. We present an alignment procedure to obtain the focus of the second FZP in coincidence with the illuminating spot and the detection pinhole. So far we have been able to demonstrate the proof-of-concept for the in-line geometry; however, ideal confocal imaging geometry can only be realised with significantly smaller spatial filter pinholes. Since we are using the second FZP as magnifying element, diameters of the detector pinhole smaller than 1 µm are required. This will provide true focus-filtered and background-suppressing imaging as known from confocal laser scanning microscopy. Furthermore, the 3D resolution of the confocal setup is significantly enhanced compared with standard STXM, since we detect a confocal volume that is ideally determined by the square of the initial point-spread function. While the lateral resolution will just marginally be affected, we expected an axial resolution limit below 100 nm for common high-resolution zone plates. Our first experimental results of cSTXM using the in-line geometry prove the applicability of this technique to typical soft X-ray microscopy specimens and the possibility to determine distinct focal planes within the recorded 3D objects.

For cSTXM in the off-line geometry (also considering true confocal conditions), the image quality strongly depends on the number of fluorescence photons (above background). Theoretical estimations as well as first experiments show that the photon efficiency of the setup installed at a bending-magnet beamline is insufficient to provide reasonable imaging so far. Considering future improvements by the use of smaller detection pinholes and technical developments that allow the detection zone plate to be brought very close to the sample, we still expect the requirement for high-brilliance radiation sources. The extremely small detection area of a zone plate compared with common large-area fluorescence detectors is impossible to compensate. Novel approaches with on-chip stacked zone plates significantly enhanced the photon efficiency of the optical system (Werner *et al.*, 2014 ▸); however, just by some percent.

Due to these limitations, further investigations will concentrate on the in-line cSTXM geometry. The use of zone plates with smaller outermost zone width enabling shorter depth of focus, an additional detection pinhole with a size of a few 100 nm and well shaped 3D standard specimens will provide detailed information on the imaging quality and resolution limits of this setup. Furthermore, we expect a strong improvement of cSTXM by the use of higher-order imaging that provides enhanced resolution in all three dimensions and lower focal length (Rehbein *et al.*, 2009 ▸; Keskinbora *et al.*, 2013 ▸). For third-order imaging with currently best resolving zone plates we can estimate a lateral resolution significantly below 10 nm and an axial resolution in the regime of some tens of nanometres. Therefore we expect cSTXM to play an important role in future high-resolution soft X-ray imaging.

## Supplementary Material

Figure S1: Scan of detector against second zone plate in off-axis CSTXM setup (710 eV).. DOI: 10.1107/S1600577514022322/pp5057sup1.pdf

## Figures and Tables

**Table 1:** Photon efficiency of cSTXM components and overall efficiency in off-axis geometry

Component	Photon efficiency	Comment
Illumination zone plate	∼0.08	First diffraction order efficiency + central stop
OSA	∼1	First-order passes when properly aligned
Sample/fluorescence yield	∼10^−2^	*L*-edges of common metals
Detection zone plate	∼6 × 10^−7^	240 µm zone plate diameter at 25 mm distance + first-order efficiency
Pinhole	∼0.1	Acts also as detection OSA
Detector	∼0.2–0.9	Type dependent
Overall efficiency	∼1 × 10^−11^ to 4 × 10^−11^	

## Footnotes

1 Supporting information for this paper is available from the IUCr electronic archives (Reference: PP5057).
