# Supplementary material for: Confocal soft X-ray scanning transmission microscopy: setup, alignment procedure and limitations
Source: J Synchrotron Radiat. 2015 Jan 1;22(Pt 1):113–8. doi: 10.1107/S1600577514022322 (PMC4785861; doi:10.1107/S1600577514022322)
Supplement: Supplementary file 1 [file s-22-00113-sup1.pdf]

JOURNAL OF SYNCHROTRON RADIATION

Volume 22 (2015)

Supporting information for article:

Confocal soft X-ray scanning transmission microscopy: setup, alignment procedure and limitations

Andreas Späth, Jörg Raabe and Rainer H. Fink

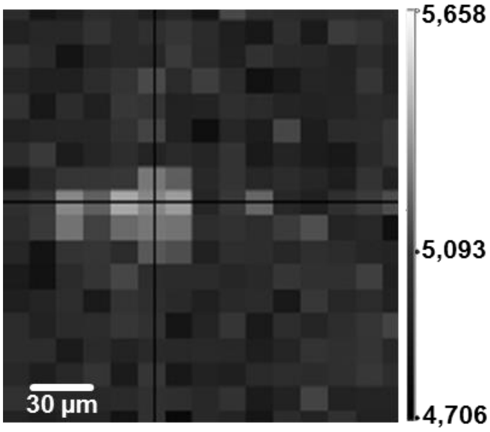

Figure S1 Scan of detector against second zone plate in off-axis CSTXM setup (710 eV). The investigated sample was a magnetite-functionalized PVA-based microballoon. Even with a dwell time of 1 s the fluorescence signal is very low compared with background intensity.
